# Correction: Comparing methylation levels assayed in GCrich regions with current and emerging methods

**DOI:** 10.1186/s12864-024-10748-7

**Published:** 2024-09-03

**Authors:** Dominic Guanzon, Jason P. Ross, Chenkai Ma, Oliver Berry, Yi Jin Liew

**Affiliations:** 1 CSIRO Health & Biosecurity, Westmead, NSW Australia; 2 Environomics Future Science Platform, CSIRO, Crawley, WA Australia; 3 Translational Extracellular Vesicles in Obstetrics and Gynae-Oncology Group, Faculty of Medicine, University of Queensland Centre for Clinical Research, The University of Queensland, Queensland, Australia

**Correction**: *BMC Genomics***25**, 741 (2024). 10.1186/s12864-024-10605-7

Following publication of the original article several errors were reported in Table 1.

In the “EPIC” column of the row “Turnaround time (from DNA extracts)” the value was given as ‘2 days’ but should be ‘3 days’.

In the “ONT” column for the row “Relative costs (per sample)” the values were given as:

$$ (for ONT Cas9)

$ (for whole genome)

The correct values are:

$$ (for ONT Cas9)

\$\$\$\$\$ (for whole genome)

The updated Table 1 with the corrected values in bold is given in this Correction article and the original article has been updated.

**Table 1:** Picking the right tool for the job

Criteria	EM-seq	WGBS	EPIC	ONT
Flexibility in DNA conversion	NEB-only	Any bisulphite conversion kit		N/A
Flexibility in library construction	NEB-only	More options	Illumina-only	ONT-only
Flexibility in sequencing	Illumina-only	Depends on library type	Illumina-only	ONT-only
Experimental complexity	Well-established protocols which can be performed by trained scientists.			Protocols actively being developed and slightly more complex.
Data analysis complexity	Robust and mature packages/pipelines available			Pipelines are still in flux
Turnaround time (from DNA extracts)	2–4 days		**3 days**	1–2 days (data is streamed)
Relative costs (per sample)	$$	$$$	$	**$$ (for ONT Cas9)** **\$\$\$\$\$ (for whole genome)**
Strengths	Cheaper than WGBS. Coverage more evenly distributed across genome. Data quality better from GC-rich loci than WGBS.	Easier to compare against publicly available data (most are WGBS/RRBS). Bisulphite conversion (without library building) cheaper than enzymatic conversion, better suited for translation into amplicon-based assays.	Very cost effective for getting a subset of methylated and biologically relevant positions across more samples. Ideal for model organisms.	Almost unbiased coverage regardless of context. Quickest turnaround time. Least affected by GC-context biases.
Weaknesses	Increased laboratory time than WGBS. Comparisons against existing data should consider readout divergences at GC-rich loci.	Coverage and methylation readouts biases very pronounced at GC-rich loci.	Not very practical for non-model organisms. Custom panels possible but less cost effective and less reliable.	Higher inputs required. Methylation data from whole genome possible, but more costly. Methylation calls are not binary, unlike bulk of existing data. Higher complexity in sequencing and in analysis.

Practical considerations involved in all four methods, as well as their relative strengths and weaknesses

