# Supplementary material for: Comparing methylation levels assayed in GC-rich regions with current and emerging methods
Source: BMC Genomics. 2024 Jul 30;25:741. doi: 10.1186/s12864-024-10605-7 (PMC11289974; doi:10.1186/s12864-024-10605-7)
Supplement: Supplementary file 2 — Supplementary Material 2 [file 12864_2024_10605_MOESM2_ESM.docx]

Comparing methylation levels assayed in GC-rich regions with current and emerging methods

Dominic Guanzon^1,3^, Jason P Ross^1^, Chenkai Ma^1^, Oliver Berry^2^, Yi Jin Liew^1,2,*^

**Affiliations**

^1^ CSIRO Health & Biosecurity, Westmead, NSW, Australia

^2^ Environomics Future Science Platform, CSIRO, Crawley, WA, Australia

^3^ UQ Centre for Clinical Research, Faculty of Medicine, The University of Queensland, QLD, Australia


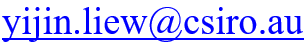
^*^ Correspondence to: (Y.J.L.)

Keywords: DNA methylation, EM-seq, WGBS, Infinium arrays, ONT, GC-rich loci

# Supplementary Figures


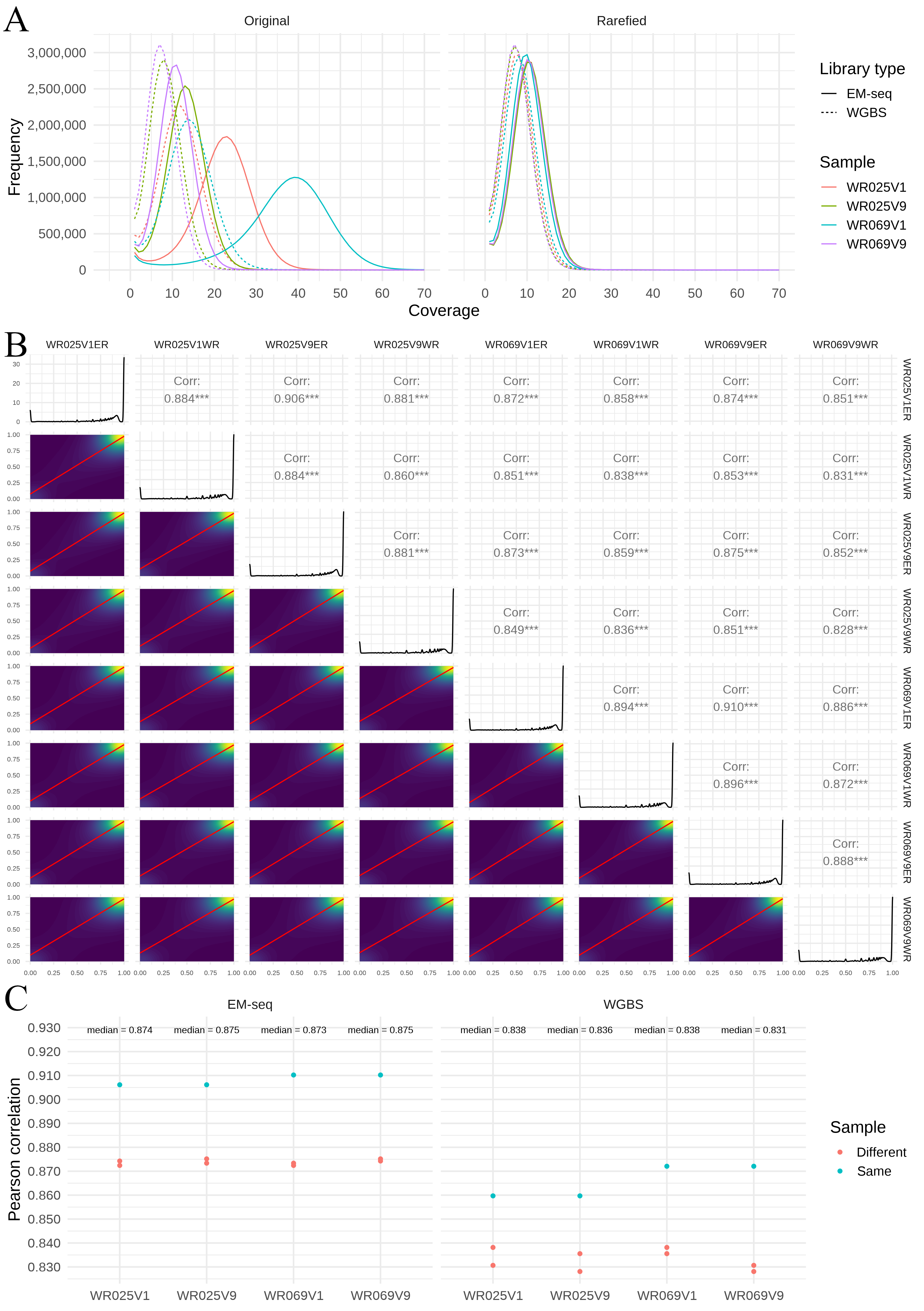


**Supplementary Figure S1. Methylation readouts from EM-seq libraries have better cross-sample correlations than WGBS libraries.** (A) Original and rarefied coverages of EM-seq libraries (solid line) compared to WGBS libraries (dashed line), coloured by sample. (B) Pearson correlation was performed on rarefied data, comparing individual EM-seq (ending “-ER”) and WGBS (ending “-WR”) samples for all 28.7 million CpG methylation beta values. The bottom left triangle contains pairwise sample comparisons represented as a 2D density plot. Red lines are lines of best fit, while the background shading indicates relative CpG density (blue: low; yellow: high). These sample comparisons are represented as Pearson correlations in the top right triangle, with *** indicating p-values < 0.001. The diagonal represents the distribution of CpG methylation beta values for individual samples. (C) Pearson correlation comparisons were separated by sample (x-axis) and grouped into EM-seq (left panel) and WGBS (right panel) library types. Comparison of samples from the same patient are coloured blue, while comparisons against different patients are coloured red.


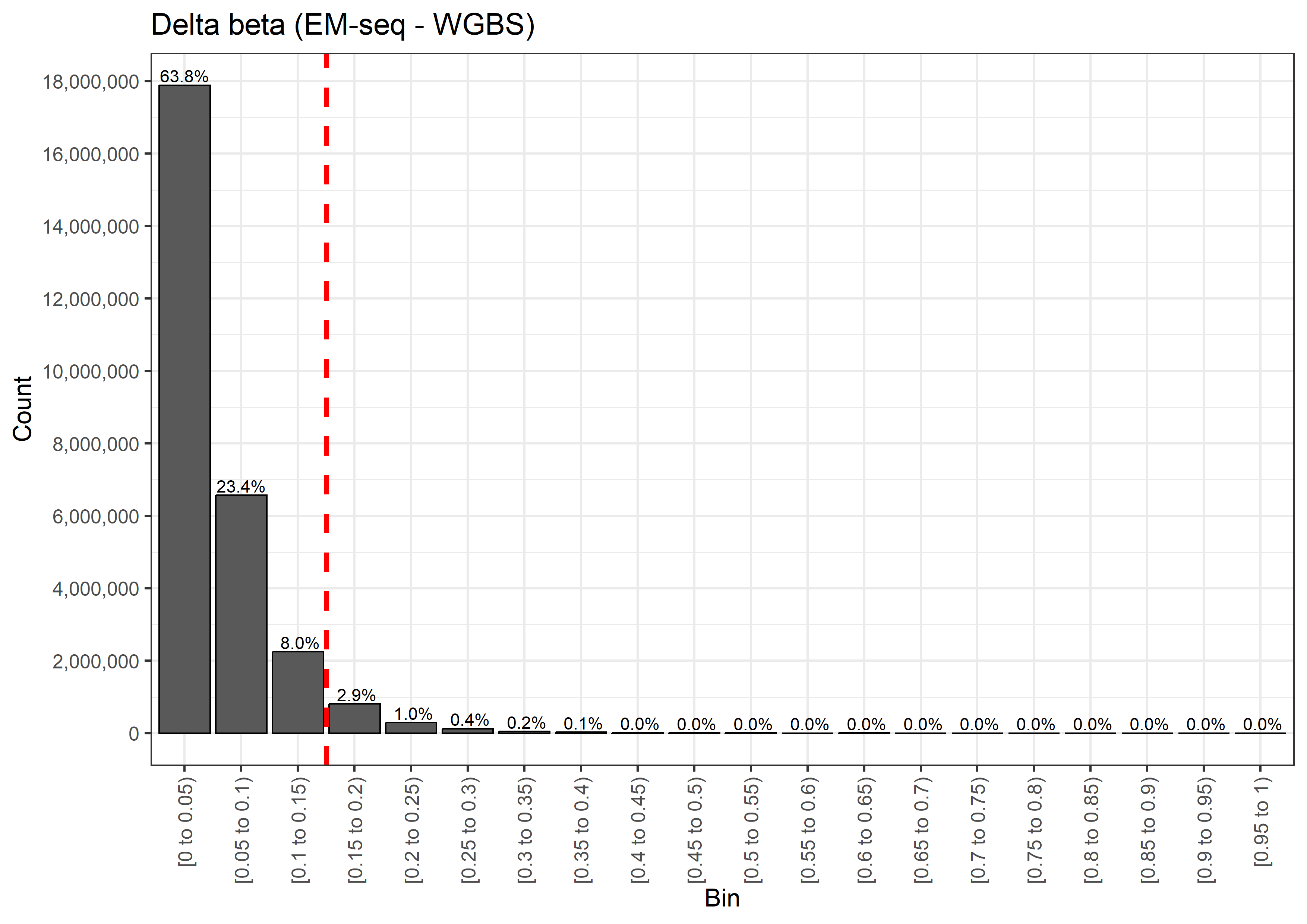


**Supplementary Figure S2. Most CpG sites (95.26%) in the human genome show minimal variation (delta beta < 0.15) between EM-seq and WGBS.** Delta beta values (EM-seq - WGBS) were calculated and grouped into bins of 0.05 increments, with the count for each bin displayed. The percentage for each bin is shown at the top of the bar plot, while the red dashed line indicates the cutoff (> 0.15 delta beta) considered to be a large difference.


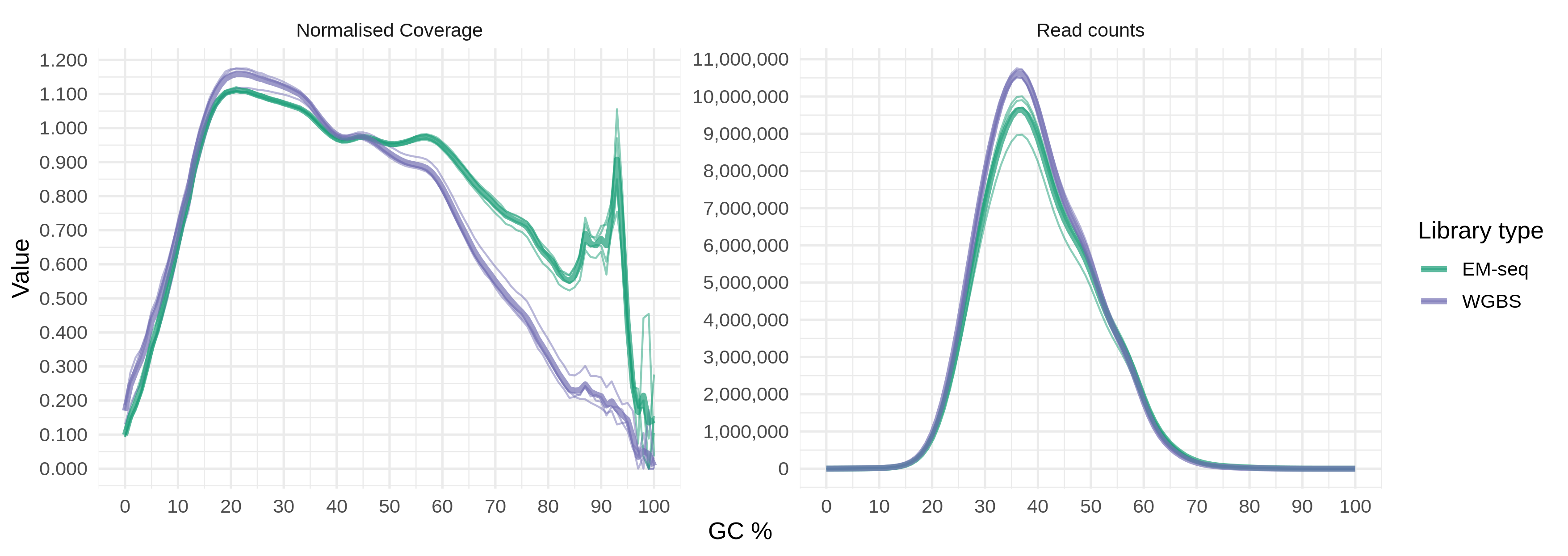


**Supplementary Figure S3. EM-seq libraries have higher coverage in GC-rich regions.** Normalised coverage (left panel) and read counts (right panel), in EM-seq libraries (green) and WGBS libraries (blue) compared to GC% in 100 base pair bins. Thick lines and thin lines represent the average and individual samples, respectively.


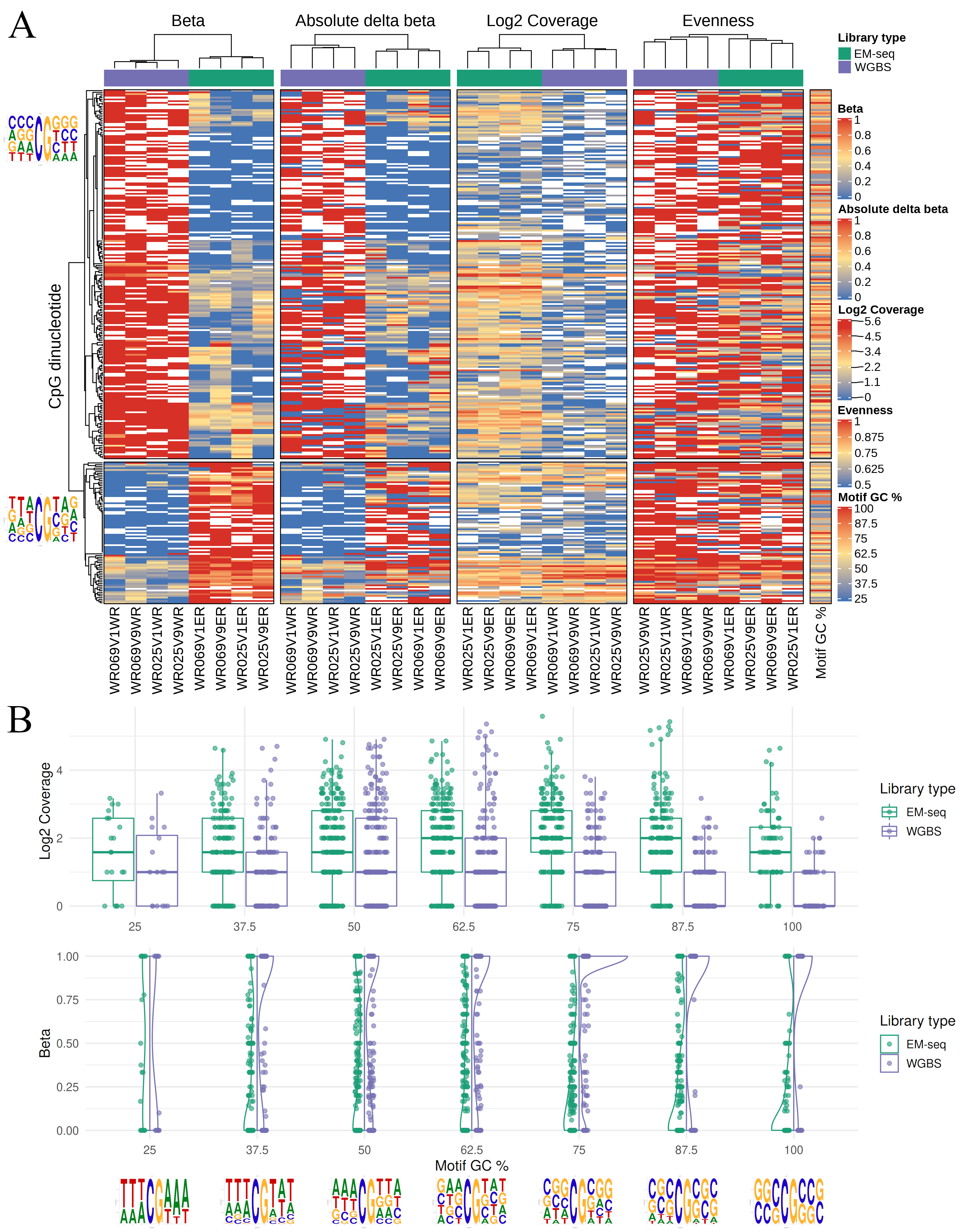


**Supplementary Figure S4. Strand-specific and motif biases associated with discordant methylation readouts after filtering for delta beta > 0.1 (EM-seq – WGBS).** (A) Beta values from 292 CpGs that were significantly different between EM-seq and WGBS libraries clustered into two groups in a heatmap. Top annotation bars represent library type, EM-seq (green) and WGBS (purple). Each column represents a different library; each row represents an individual CpG site. Dinucleotides with higher betas in WGBS are above, while those higher in EM-seq are below. Overall and strand-specific methylation levels are represented by “Beta” and “Absolute delta beta” respectively; overall and strand-specific coverages are represented by “Log2 coverage” and “Evenness” respectively. Sequence logos of 8 bp CpG motifs for both groups are placed beside the left dendrogram, while GC% of the motifs are on the rightmost column (“Motif GC%”). White boxes in the heatmap represent missing values. (B) Discordant CpGs were initially split by GC% of 8 bp motifs and subsequently by library type, EM-seq (green) and WGBS (purple). Individual bins were plotted against coverage (top panel) and beta (bottom panel). Sequence logos for each GC% bin is shown below the x-axis.


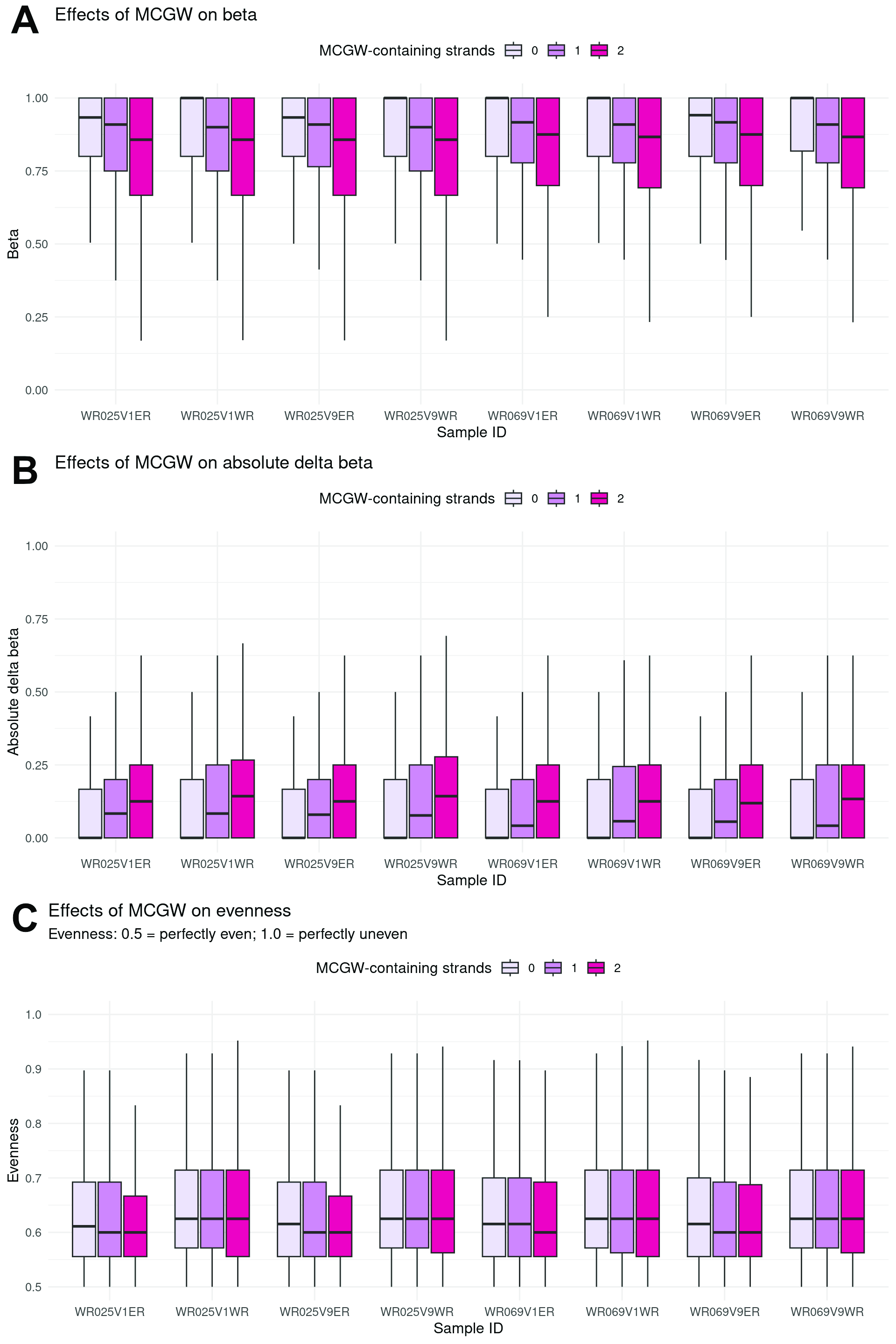


**Supplementary Figure S5. Methylation readouts binned by MCGW status for all eight samples.** All CpGs with sufficient coverage (≥ 5; > 22 million dinucleotides for all samples) were categorised by MCGW-containing strands. As MCGW is not a palindromic motif, out of the overall 16 NCGN possibilities, 9 contexts have neither strand with MCGW, 6 have a single strand with MCGW, and 1 where both strands are MCGW (ACGT/ACGT). If EM-seq libraries had biased beta readouts linked with MCGW status, the distribution of (A) beta values, (B) absolute delta beta values and (C) evenness would be different in samples with “-ER” (EM-seq libraries) than those with “-WR” (WGBS). In all three cases, MCGW status has little-to-no effect on those values.


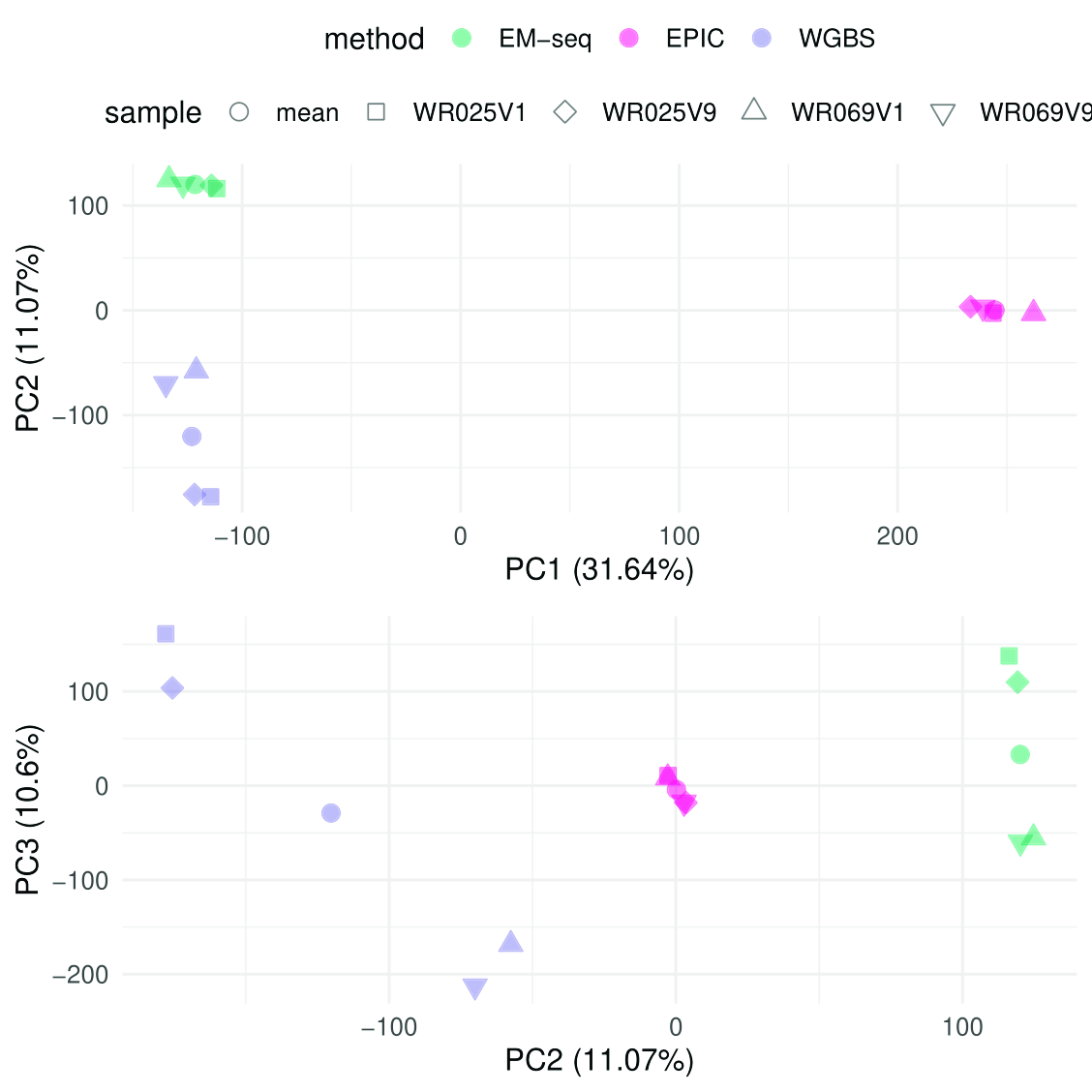


**Supplementary Figure S6. Principal components analysis of per-base methylation levels of four samples across EM-seq, WGBS and EPIC.** Variation in the first two principal axes were mainly driven by methodological differences: EPIC and short-read methods occupying separate ends of the first principal component, while the short-read methods separate well on the second principal component. The third principal component (with explained variance value just under the second component) separated by biological sample origin: the four-sided shapes (WR025) and the three-sided ones (WR069) tend to occupy opposite ends. This separation was obvious for the short-read methods but not for EPIC, in line with the lowest variance amongst the EPIC datasets. WGBS had the highest variance. To facilitate downstream comparisons, per-position, per-method mean methylation levels were calculated (filled round points) and included in the plot to confirm they are located amongst the four constituent replicates.


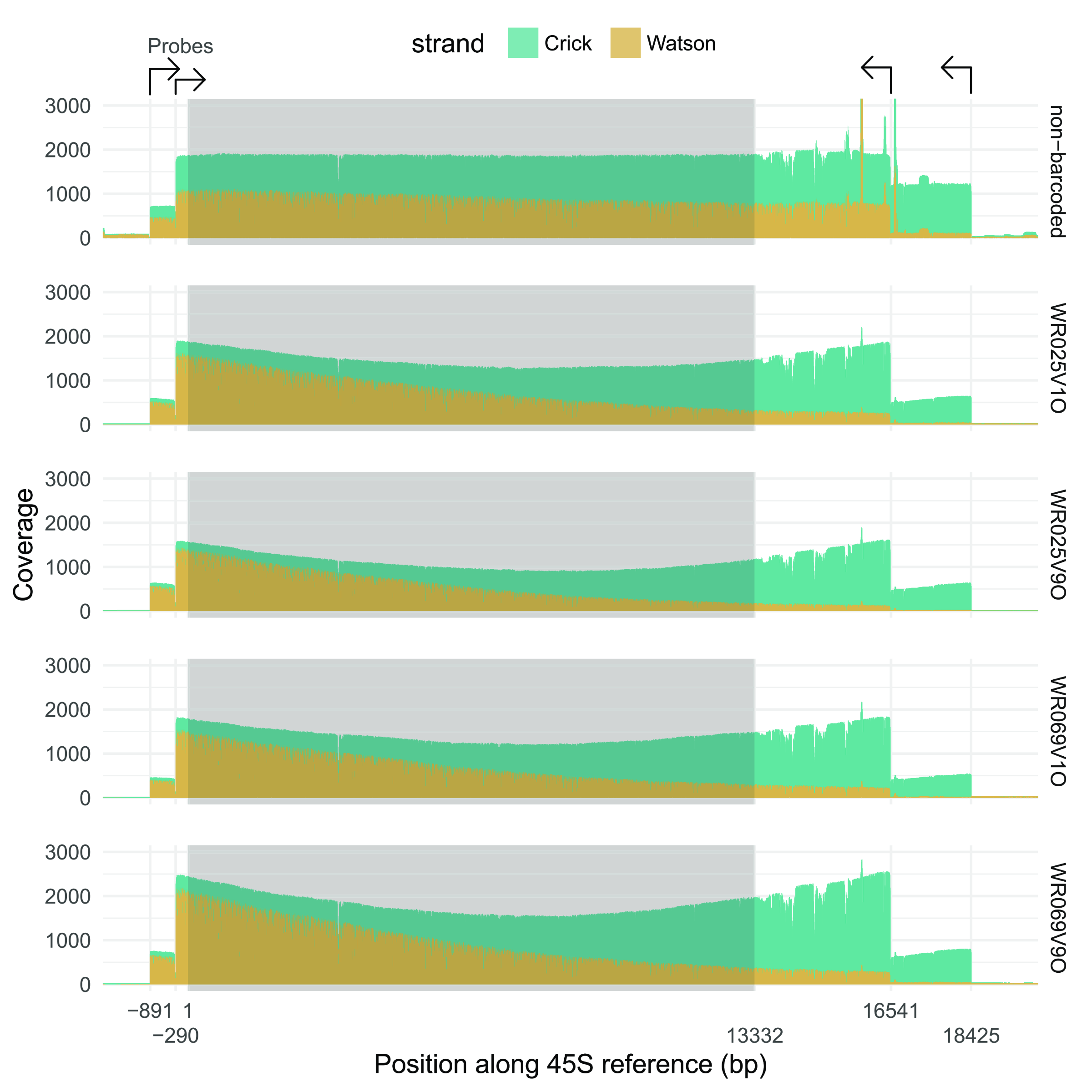


**Supplementary Figure S7. Coverage of mapped reads along the 45S reference used in this experiment.** Coverages were plotted separately for each sample (and for reads that were not barcoded). Shaded region represents the transcribed 45S regions (1–13,332 bp). The x-axis positions of -891, -290, 16,451 and 18,425 bp represent the 3’ positions of the probes used in this experiment, and the direction of the probes are indicated at the top of the plot. As Cas9 makes targeted blunt-end cuts in the DNA, the length of the read can be shorter than the gap between cut sites if the other end was sheared/degraded through normal means. This results in a typical U-shaped coverage plot, seen in all barcoded samples. The flatter profile for non-barcoded reads is not well understood (ONT, personal communication).


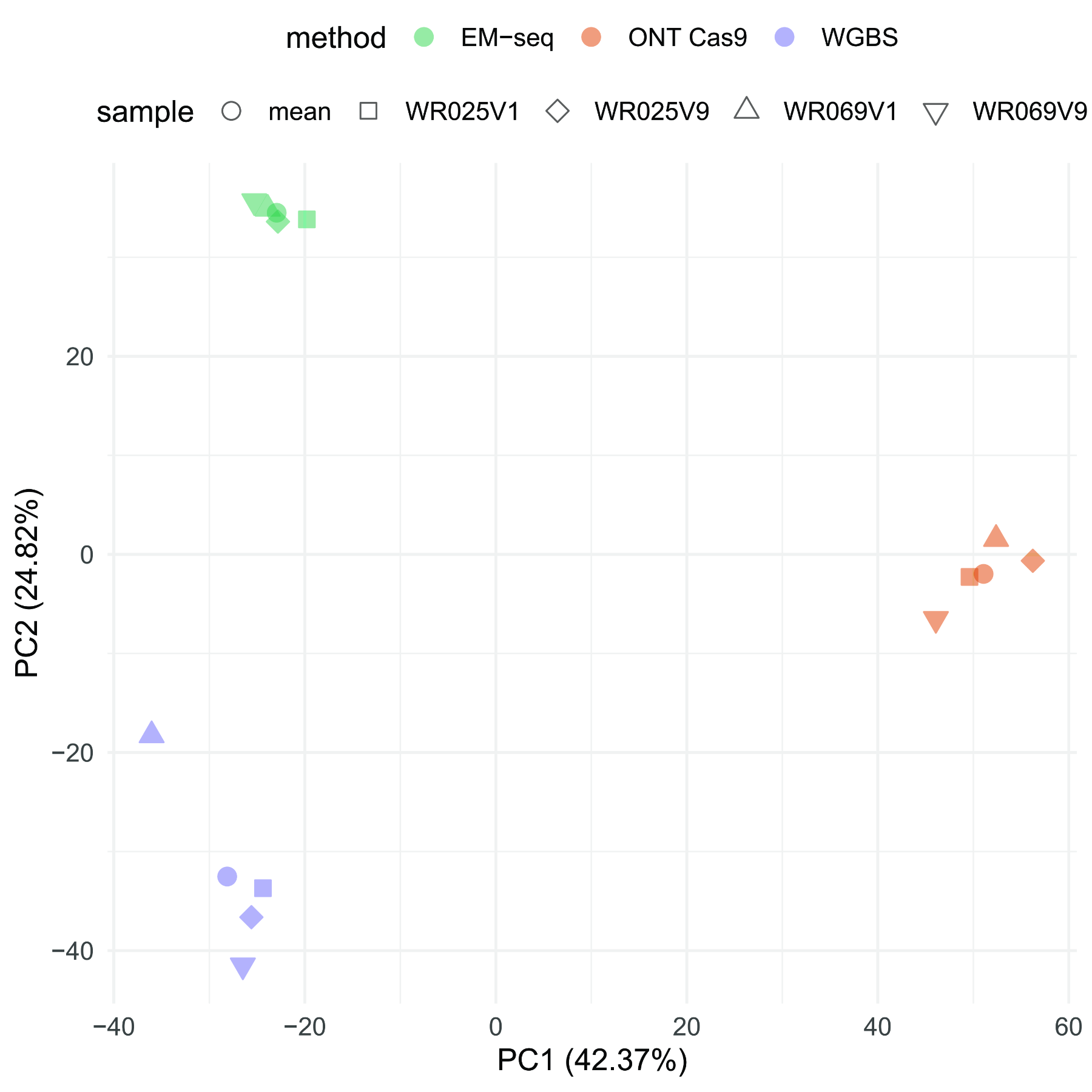


**Supplementary Figure S8. Principal components analysis of per-base methylation levels of four samples across EM-seq, WGBS and ONT Cas9.** Variation in the first two principal axes were mainly driven by methodological differences. Replicates within EM-seq datasets had the lowest variation; WGBS highest. To facilitate downstream comparisons, per-position, per-method mean methylation levels were calculated (filled round points) and included in the plot to confirm they are located amongst the four constituent replicates.


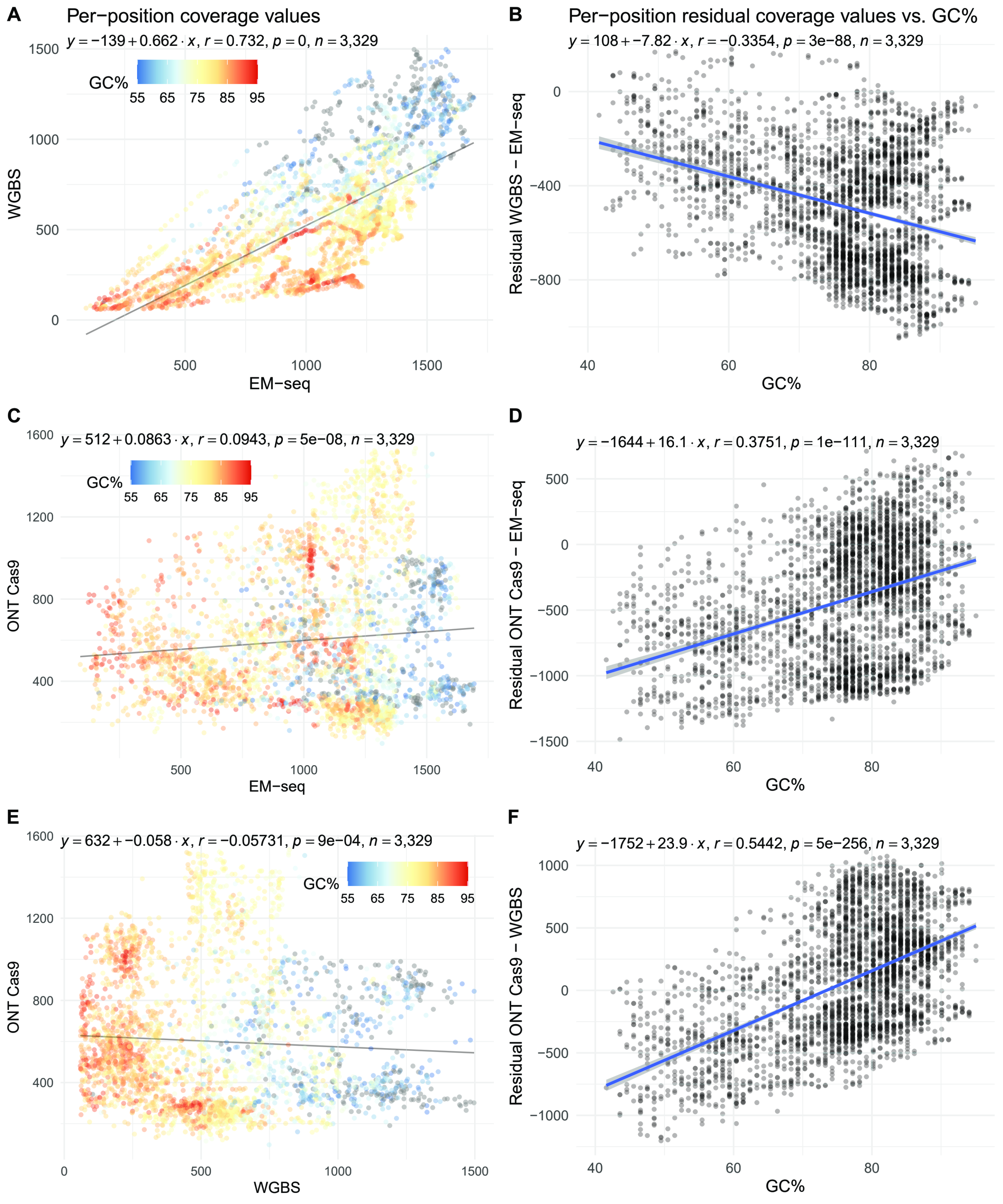


**Supplementary Figure S9. Pairwise comparisons of per-position coverage values from EM-seq, WGBS and ONT Cas9. A** and **B** are for EM-seq vs. WGBS; **C** and **D** are for ONT Cas9 vs. EM-seq; while **E** and **F** are for ONT Cas9 vs. WGBS. Each point in the plots on the left (**A**, **C** and **E**) represent a single cytosine, and its position on the plot corresponds to the mean coverage value assayed using the method labelled on the axes. It is further coloured by the GC% of its local sequence context (±50 bp) to demonstrate context-dependent biases in coverages. For each point, the residual (differences in coverage values) was computed and plotted against the same GC% value on the right (**B**, **D** and **F**). Coverage values between the short-read methods were better correlated (*r^2^* = 0.54) than either method against ONT Cas9 (*r^2^* < 0.01). EM-seq outperforms WGBS in all available GC% contexts, especially at high GC% contexts (> 75%).
